# Adolescent mental health utilization, virtual care, and community support: evidence from 2019 to 2022

**DOI:** 10.3389/fpubh.2025.1559511

**Published:** 2025-07-28

**Authors:** William Youkang Zhou, Luisa Franzini, Arturo Vargas Bustamante

**Affiliations:** ^1^Walt Whitman High School, Bethesda, MD, United States; ^2^Department of Health Policy and Management, School of Public Health, University of Maryland, College Park, MD, United States; ^3^Department of Health Policy and Management, Fielding School of Public Health, University of California, Los Angeles, Los Angeles, CA, United States

**Keywords:** adolescent, mental health services, race and ethnicity, community support, telemedicine, utilization, access to care

## Abstract

**Objective:**

This study examined racial and ethnic disparities in mental health service use, social support, and telemedicine access among U.S. adolescents between 2019 and 2022.

**Methods:**

We analyzed nationally representative data from 2019 to 2022 National Health Interview Survey (NHIS) Sample Child Interview, focusing on adolescents aged 12–17. Multivariate logistic regression models with survey weights were used to assess disparities in outcomes by race and ethnicity.

**Results:**

From 2019 to 2022, despite rising mental health needs, Black, Hispanic, and Asian adolescents were significantly less likely than White peers to take prescription medications (7–12 percentage points lower, *p* < 0.001), receive therapy (4–12 percentage points lower, *p* < 0.001), or receive both treatments (4–7 percentage points lower, *p* < 0.001). Hispanic and Asian adolescents were also 9 and 15 percentage points less likely (*p* < 0.001), respectively, to report receiving community support, while Black and Asian adolescents were 8 and 6 percentage points less likely (*p* < 0.001), respectively, to have had a virtual healthcare appointment.

**Conclusions:**

Access to mental health services, virtual care, and community support remains disproportionately limited for racial and ethnic minority adolescents, even as overall mental health needs have worsened across all groups during the COVID-19 pandemic. The underuse of virtual care and community support among Hispanic and Asian adolescents underscores the urgent need for culturally responsive strategies to promote accessible and personalized mental health care for all adolescents.

## Introduction

Adolescent mental health issues are an increasing public health concern. In 2021, approximately 3.7 million U.S. adolescents aged 12–17 experienced at least one major depressive episode in the past year, representing 14.7% of this age group (1). Adolescence is a crucial stage of development, as mental health challenges during this period can lead to more severe mental health issues in adulthood (2). Racial and ethnic disparities in mental health care access have been well-documented. Between 2010 and 2017, studies showed that Black and Hispanic adolescents faced significant disparities in psychotropic medication use compared to White adolescents, and this gap worsened over time (3). The COVID-19 pandemic and associated quarantines exacerbated vulnerability to mental health problems among adolescents overall (4, 5). However, there is limited recent evidence on patterns of mental healthcare utilization since the pandemic.

Emerging research highlights potential strategies to mitigate the adolescent mental health crisis. For instance, social support has been shown to positively impact mental health outcomes (6). In addition, telemedicine and virtual interventions have demonstrated effectiveness in improving behavioral health (7–9). While telehealth services expanded significantly during the pandemic, evidence regarding telemedicine use among adolescents remains limited (7).

The objective of this study is to investigate racial and ethnic disparities in mental health service utilization, social support, and telemedicine use among U.S. adolescents from 2019 to 2022. We hypothesize that racial and ethnic minority adolescents continue to face significantly lower odds of accessing mental health services, while mental health challenges have worsened across all racial and ethnic groups since the onset of the COVID-19 pandemic.

To guide our model specification and test this hypothesis, we applied Andersen's Behavioral Model of Health Services Use (8). This framework covers three domains: predisposing factors (e.g., race/ethnicity, age), enabling factors [e.g., socioeconomic status (SES), insurance coverage], and need-based factors (e.g., health conditions). These domains are particularly relevant for understanding disparities in adolescent mental healthcare utilization. For example, compared to their White counterparts, Black and Hispanic adolescents are more likely to live in households with lower income, lower parental education, and limited health insurance coverage (9, 10). These SES-related barriers may hinder access to mental health services and contribute to delays in recognizing and responding to mental health issues (9, 11).

In addition to mental healthcare, community-level social support is a key protective factor for adolescent mental health. Research has shown that strong social support is associated with lower rates of depressive symptoms and greater life satisfaction among youth (12). However, the COVID-19 pandemic significantly limited social interaction, and adolescents without access to adequate support, experienced worse mental health outcomes, including signs of accelerated brain aging (4, 13). Moreover, cultural factors may influence how adolescents engage with social support networks. For instance, Hispanic and Asian American adolescents may be less likely than their White peers to seek or receive community-based support due to cultural norms, stigma, or systemic barriers (14). Therefore, we further hypothesize that the association between community social support and mental health outcomes differs by race and ethnicity. Our study aligns with national public health priorities such as Healthy People 2030, which emphasizes the importance of reducing disparities and addressing social determinants of health (15).

## Materials and methods

Our study utilized data from the National Health Interview Survey (NHIS) Sample Child Interview, focusing on adolescents aged 12–17. The NHIS Sample Child Survey conducts annual interviews with a nationally representative sample of households, collecting health information about children from their parents or guardians. For this study, we used data collected between 2019 and 2022. The outcome measures, including mental healthcare utilization, span across each survey year (16, 17).

### Outcome measures

This study focused on three primary outcome domains: mental health service utilization, community support, and telemedicine use. Mental health service utilization was measured based on whether the adolescent: (a) took prescription medication for issues related to emotions, concentration, behavior, or mental health; (b) received counseling or therapy from a mental health professional (e.g., psychiatrist, psychologist, psychiatric nurse, or clinical social worker); and (c) received both therapy and medication in the past year. These three outcomes were available in interviews conducted from 2019 to 2022.

Community support was assessed using two measures: (1) whether the adolescent received the social and emotional support they needed, and (2) whether the adolescent had at least one adult, other than a parent or household member, in their school, neighborhood, or broader community who made a positive and meaningful difference in their life. These variables were coded as 1 if the respondent answered “always,” and 0 otherwise. These outcomes were available in interviews conducted from 2021 to 2022.

Telemedicine use was measured by whether the adolescent had a video or phone appointment with a doctor, nurse, or other health professional in the past 12 months. This outcome was available in interviews conducted from 2020 to 2022.

### Independent measures

The key independent variables in this study were informed by Andersen's Behavioral Model of Health Services Use and prior literature on adolescent mental health. The primary variable of interest was race and ethnicity, categorized as non-Hispanic White (White), non-Hispanic Black (Black), non-Hispanic Asian (Asian), and Hispanic. Additional covariates included age, sex, nativity (whether the adolescent was born in the U.S.), type of health insurance, self-reported general health, family income, parental highest level of education, parental marital status, and urban vs. rural residence. We also included year indicators to adjust for temporal trends over the study period.

To account for health needs, we controlled for adolescents' self-reported general health status. To further capture mental health needs, we included a parent-reported measure of depressive frequency, based on the NHIS survey question: “How often does [the adolescent] seem very sad or depressed? Would you say daily, weekly, monthly, a few times a year, or never?”

### Analysis

This study focused on mental health service utilization among adolescents. First, we plotted the parent-reported frequency of depressive symptoms by race and ethnicity from 2019 to 2022 as a proxy for trends in mental health needs. We then summarized the sociodemographic characteristics of adolescents by racial and ethnic groups.

To examine associations between race/ethnicity and each outcome of interest, we conducted multivariate logistic regression analyses, controlling all covariates described above. We report average marginal effects (AMEs) to aid interpretation. All analyses incorporated NHIS survey weights to ensure nationally representative estimates.

We also conducted sensitivity analyses to assess the robustness of our findings. We excluded the parent-reported frequency of feeling depressed, recognizing that parental perception may not accurately reflect the adolescent's internal experience (see Appendix 1). In addition, screen time has been identified in prior research as a factor associated with poorer mental health outcomes (18). We included a control for screen time, defined as whether the adolescent spent more than two hours per day on a device for social or entertainment purposes (e.g., playing games, watching videos, using social media, or browsing the internet; see Appendix 2) (12). Our findings remained consistent across these alternative model specifications.

All analyses were conducted using Python, specifically the Pandas (version 2.0.3) and Statsmodels packages. Survey weights were applied throughout to maintain national representativeness. This study used publicly available, de-identified NHIS data and was therefore exempt from human subject research review.

## Results

We first analyzed three main utilization measures using data from the NHIS Sample Child files from 2019 to 2022, focusing on adolescents aged 12–17. The pooled sample across 4 years included adolescents identified as White, Black, Hispanic, or Asian, with a total sample size of *N* = 10,974. After excluding 259 cases (approximately 2%) due to missing values on covariates, the final analytic sample was *N* = 10,715, consisting of 5,981 White, 1,186 Black, 2,804 Hispanic, and 744 Asian adolescents.

The outcomes “emotional/social support available” and “presence of community support” were available only in 2021 and 2022, with an initial sample of *N* = 5,532. After accounting for missing data, the final analytic samples were *N* = 5,393 for emotional/social support and *N* = 5,403 for community support, with approximately 2% missingness in each.

The outcome “had any virtual care” was available from 2020 to 2022, with an initial sample of *N* = 7,733. The final analytic sample for telemedicine use was *N* = 6,117, based on whether the parent reported that the adolescent had any healthcare appointment during the reference period.

**Figure 1:**
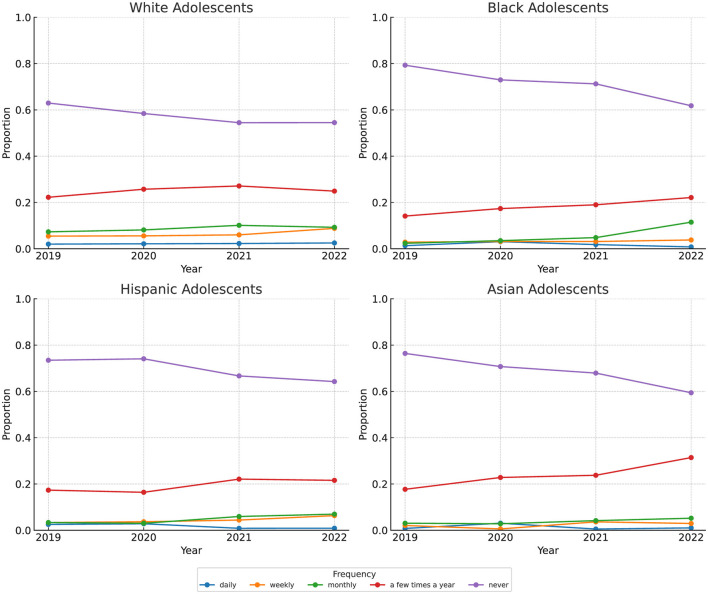
How often seems sad or depressed. Data: Our study used the 2019–2022 National Health Interview Survey's (NHIS) Sample Child Interview and focused on adolescents aged 12–17. How often an adolescent seemed very sad or depressed was measured on a scale from 1 to 5, with 1 being daily, 2 weekly, 3 monthly, 4 a few times a year, and 5 never.

Figure 1 shows the frequency of feeling depressed among adolescents during this period, disaggregated by race and ethnicity. Among White adolescents, the percentage reporting never feeling depressed dropped from 63% to 55%. This decline was even more pronounced among Black adolescents (79%−62%), Asian adolescents (76%−59%), and Hispanic adolescents (74%−64%). Meanwhile, the share of Black, Hispanic, and Asian adolescents reporting feeling depressed a few times a year rose by 8, 5, and 4%, respectively, compared to a 3% increase among White adolescents. Additionally, Black adolescents reporting feeling depressed monthly increased dramatically, from 2% to 11%. Similarly, the proportion of Hispanic and Asian adolescents reporting feeling depressed monthly doubled, rising from 3% to 7%.

Table 1 shows that 12% of adolescents received therapy from a mental health professional, 15% took medication for their mental health, 8% received both therapy and medication, 77% always had social support, 89% received community support, and 20% had a virtual general health appointment. Variations by race and ethnicity were observed. Compared to White adolescents, Black, Hispanic, and Asian adolescents had lower frequencies of taking mental health medication, receiving therapy, or utilizing both therapy and medication. For instance, 16% of White adolescents reported taking medication, compared to 8% of Black, 7% of Hispanic, and 3% of Asian adolescents (*p* < 0.001). Hispanic and Asian adolescents were also significantly less likely to have community support compared to White and Black adolescents (*p* < 0.001). Furthermore, approximately 22% of White adolescents reported having a virtual medical appointment, followed by Hispanic (19%), Asian (16%), and Black adolescents (13%) (*p* < 0.01).

**Table 1:** Sample characteristics of adolescents by race and ethnicity.

	**All adolescents**		**White adolescents**		**Black adolescents**			**Hispanic adolescents**			**Asian adolescents**		
**Sample size**	***N*** = **10,715**		***N*** = **5,981**		***N*** = **1,186**			***N*** = **2,804**			***N*** = **744**		
	**Mean**	**std**	**Mean**	**std**	**Mean**	**std**	***p***	**Mean**	**std**	***p***	**Mean**	**std**	***p***
**Outcome measures**													
Took medication for mental health	0.12	0.33	0.16	0.37	0.08	0.27	< 0.001	0.07	0.26	< 0.001	0.03	0.18	< 0.001
Had mental health therapy	0.15	0.36	0.19	0.39	0.11	0.31	< 0.001	0.13	0.33	< 0.001	0.05	0.22	< 0.001
Had medication and therapy	0.08	0.27	0.10	0.30	0.05	0.22	< 0.001	0.05	0.22	< 0.001	0.02	0.14	< 0.001
Had any virtual care^a^	0.20	0.40	0.22	0.42	0.13	0.34	< 0.001	0.19	0.39	0.004	0.16	0.37	0.002
Emotional/social support available^b^	0.77	0.42	0.78	0.41	0.82	0.38	0.02	0.76	0.43	0.10	0.72	0.45	0.01
Presence of community support^c^	0.89	0.31	0.95	0.21	0.90	0.29	< 0.001	0.79	0.40	< 0.001	0.79	0.40	< 0.001
**Independent variables**													
Female	0.48	0.50	0.49	0.50	0.48	0.50	0.56	0.48	0.50	0.22	0.48	0.50	0.71
Age (years)	14.62	1.70	14.67	1.69	14.61	1.72	0.22	14.54	1.68	0.00	14.54	1.72	0.04
US-born	0.94	0.24	0.98	0.14	0.95	0.21	< 0.001	0.91	0.29	< 0.001	0.72	0.45	< 0.001
**Self-reported health**													
Excellent or very good health	0.84	0.36	0.88	0.32	0.81	0.39	< 0.001	0.76	0.43	< 0.001	0.88	0.32	0.88
Good health	0.12	0.33	0.09	0.29	0.15	0.36	< 0.001	0.19	0.39	< 0.001	0.10	0.30	0.34
Fair or poor health	0.03	0.17	0.02	0.16	0.03	0.18	0.12	0.05	0.22	< 0.001	0.02	0.13	0.14
Never felt depressed	0.62	0.49	0.57	0.50	0.72	0.45	< 0.001	0.68	0.47	< 0.001	0.69	0.46	< 0.001
**Family income**													
< 100% federal poverty line (FPL)	0.11	0.32	0.06	0.24	0.22	0.41	< 0.001	0.19	0.39	< 0.001	0.08	0.27	0.12
100–200% FPL	0.20	0.40	0.13	0.34	0.27	0.45	< 0.001	0.32	0.47	< 0.001	0.17	0.37	0.02
200–400% FPL	0.30	0.46	0.31	0.46	0.29	0.46	0.34	0.31	0.46	0.83	0.28	0.45	0.07
Above 400% FPL	0.38	0.49	0.50	0.50	0.22	0.41	< 0.001	0.19	0.39	< 0.001	0.48	0.50	0.48
**Highest education of parents**													
No high school	0.07	0.25	0.02	0.14	0.05	0.22	< 0.001	0.18	0.39	< 0.001	0.03	0.18	0.01
High school	0.30	0.46	0.24	0.43	0.42	0.49	< 0.001	0.41	0.49	< 0.001	0.16	0.36	< 0.001
College	0.40	0.49	0.45	0.50	0.36	0.48	< 0.001	0.30	0.46	< 0.001	0.43	0.50	0.31
Graduate school	0.22	0.41	0.27	0.44	0.16	0.37	< 0.001	0.10	0.30	< 0.001	0.35	0.48	< 0.001
Parent marital status: married	0.61	0.49	0.68	0.47	0.32	0.47	< 0.001	0.54	0.50	< 0.001	0.80	0.40	< 0.001
**Insurance**													
Uninsured	0.05	0.22	0.03	0.18	0.04	0.19	0.33	0.10	0.29	< 0.001	0.03	0.17	0.82
Private insurance	0.62	0.48	0.75	0.43	0.44	0.50	< 0.001	0.41	0.49	< 0.001	0.72	0.45	0.05
Public insurance	0.30	0.46	0.19	0.39	0.49	0.50	< 0.001	0.47	0.50	< 0.001	0.24	0.42	< 0.001
Other insurance	0.03	0.16	0.03	0.17	0.04	0.18	0.29	0.02	0.15	0.13	0.02	0.13	0.04
Rural	0.14	0.35	0.20	0.40	0.09	0.28	< 0.001	0.06	0.24	< 0.001	0.02	0.15	< 0.001

Mean unit 100% unless indicated otherwise. std, standard deviation. *p*-value, white adolescents served as the reference group. Data, our study used the 2019–2022 National Health Interview Survey's (NHIS) Sample Child Interview and focused on adolescents aged 12–17.

^a^The “Emotional/Social Support” measure was available only in 2021 and 2022. The total analytic sample was *N* = 5,393, including 2,864 White, 606 Black, 1,499 Hispanic, and 424 Asian adolescents.

^*b*^The “Community Support” measure was also available only in 2021 and 2022. The total sample was *N* = 5,372, including 2,858 White, 604 Black, 1,487 Hispanic, and 423 Asian adolescents.

^c^The “Had Virtual Medical Appointment” measure was available beginning in 2020. The total sample for this measure was *N* = 6,117, including 3,296 White, 671 Black, 1,667 Hispanic, and 483 Asian adolescents.

After controlling for confounding variables, Table 2 shows significant racial and ethnic disparities in mental health treatment. Compared to White adolescents, Black (OR = 0.45, *p* < 0.001), Hispanic (OR = 0.44, *p* < 0.001), and Asian adolescents (OR = 0.17, *p* < 0.001) were less likely to use prescription drugs for mental health. Similarly, Black (OR = 0.60, *p* < 0.001), Hispanic (OR = 0.58, *p* < 0.001), and Asian adolescents (OR = 0.16, *p* < 0.001) were less likely to receive therapy, and all three groups were significantly less likely to receive both treatments (OR = 0.55, 0.58, and 0.20, respectively; *p* < 0.001). Additionally, adolescents with better reported general and mental health and those without insurance were less likely to access mental health treatment.

**Table 2:** Racial and ethnic differences in mental health care utilization among U.S. adolescents.

	**Took medication for mental health**				**Had mental health therapy**				**Had medication and therapy**			
	**OR**	**95% CI**		***p***	**OR**	**95% CI**		***p***	**OR**	**95% CI**		***p***
**Race and ethnicity**												
White	reference				reference				reference			
Black	0.45	0.33	0.61	< 0.001	0.60	0.45	0.80	< 0.001	0.55	0.38	0.80	< 0.001
Hispanic	0.44	0.35	0.56	< 0.001	0.70	0.58	0.86	< 0.001	0.58	0.43	0.77	< 0.001
Asian	0.17	0.11	0.28	< 0.001	0.23	0.16	0.34	< 0.001	0.20	0.11	0.38	< 0.001
Female	0.72	0.61	0.84	< 0.001	1.06	0.92	1.23	0.42	0.91	0.75	1.10	0.34
Age	1.05	1.01	1.10	0.02	1.01	0.97	1.06	0.54	1.08	1.02	1.14	0.01
US-born	1.31	0.79	2.19	0.30	1.25	0.84	1.86	0.27	1.13	0.66	1.94	0.65
**Self-reported health**												
Fair and poor health	reference				reference				reference			
Excellent or very good health	0.25	0.18	0.35	< 0.001	0.32	0.23	0.45	< 0.001	0.24	0.17	0.34	< 0.001
Good health	0.48	0.33	0.71	< 0.001	0.59	0.42	0.84	< 0.001	0.48	0.33	0.72	< 0.001
Never felt depressed	0.27	0.23	0.31	< 0.001	0.16	0.14	0.19	< 0.001	0.16	0.12	0.20	< 0.001
**Family income**												
< 100% FPL	reference				reference				reference			
100%−200% FPL	1.00	0.73	1.37	0.98	1.07	0.81	1.42	0.64	0.97	0.67	1.43	0.89
200%−400% FPL	1.02	0.74	1.40	0.91	1.32	0.99	1.76	0.06	1.09	0.75	1.59	0.65
Above 400% FPL	1.21	0.86	1.68	0.27	1.60	1.17	2.20	< 0.001	1.46	0.96	2.21	0.08
**Highest education of parents**												
No high school	reference				reference				reference			
High school	1.18	0.81	1.73	0.38	0.87	0.62	1.20	0.39	0.96	0.60	1.53	0.85
College	1.35	0.94	1.94	0.11	1.05	0.75	1.47	0.78	1.17	0.73	1.88	0.51
Graduate school	1.43	0.97	2.10	0.07	1.24	0.87	1.76	0.23	1.40	0.86	2.27	0.17
Parent marital status: married	0.77	0.64	0.92	< 0.001	0.63	0.53	0.74	< 0.001	0.66	0.52	0.83	< 0.001
**Insurance**												
Uninsured	reference				reference				reference			
Private insurance	2.40	1.35	4.28	< 0.001	2.45	1.54	3.90	< 0.001	4.33	2.13	8.81	< 0.001
Public insurance	3.07	1.72	5.47	< 0.001	3.46	2.17	5.53	< 0.001	6.02	2.89	12.54	< 0.001
Other insurance	2.61	1.30	5.22	0.01	2.56	1.38	4.74	< 0.001	4.03	1.75	9.28	< 0.001
Rural	1.13	0.89	1.44	0.31	0.84	0.66	1.05	0.13	0.96	0.69	1.33	0.81
**Year**												
2019	reference				reference				reference			
2020	1.09	0.87	1.35	0.46	1.14	0.92	1.41	0.22	1.14	0.85	1.53	0.37
2021	0.87	0.71	1.06	0.17	1.03	0.86	1.23	0.74	0.80	0.62	1.02	0.07
2022	1.11	0.92	1.35	0.27	1.33	1.12	1.59	< 0.001	1.19	0.94	1.51	0.15
Constant	0.16	0.05	0.47	< 0.001	0.28	0.11	0.73	0.01	0.04	0.01	0.17	< 0.001

Data, Our study used the 2019–2022 National Health Interview Survey's (NHIS) Sample Child Interview and focused on adolescents aged 12 to 17. “Emotional/social support” measure was only available in 2021 and 2022, and “had virtual medical appointment” measure was only available since 2020.

The sample size of the regressions *N* = 10,715, and weighted *N* = 92,789,424.

Compared to White adolescents, Hispanic (OR = 0.34, *p* < 0.001) and Asian adolescents (OR = 0.23, *p* < 0.001) were significantly less likely to receive community support (Table 3). Additionally, Asian adolescents were less likely to receive social or emotional support compared to White adolescents (OR = 0.71, *p* < 0.05). No significant differences were observed in receiving social or community support between White and Black adolescents. However, Black (OR = 0.59, *p* < 0.01) and Asian adolescents (OR = 0.59, *p* < 0.01) were significantly less likely than White adolescents to have a virtual appointment.

**Table 3:** Outcomes of virtual care and social support among US adolescents.

	**Had any virtual care**				**Emotional/social support available**				**Presence of community support**			
**Sample size**	***N*** = **6,117**				***N*** = **5,393**				***N*** = **5,372**			
	**OR**	**95% CI**		***p***	**OR**	**95% CI**		***p***	**OR**	**95% CI**		***p***
**Race and ethnicity**												
White	reference				reference				reference			
Black	0.59	0.41	0.86	0.01	1.22	0.91	1.65	0.19	0.69	0.47	1.03	0.07
Hispanic	0.89	0.72	1.09	0.25	0.86	0.69	1.07	0.16	0.34	0.25	0.45	< 0.001
Asian	0.59	0.40	0.86	0.01	0.71	0.52	0.98	0.04	0.23	0.15	0.34	< 0.001

Data: Our study used the 2019–2022 National Health Interview Survey's (NHIS) Sample Child Interview and focused on adolescents aged 12–17. “Emotional/social support” measure was only available in 2021 and 2022, and “had virtual medical appointment” measure was only available since 2020.

The sample size of the regression virtual care is *N* = 6,117, and weighted *N* = 58,080,75; social support availability *N* = 5,393, and weighted *N* = 46,652,178; community support presence *N* = 5,372, and weighted *N* = 46,459,936.

Other covariates were controlled.

Table 4 reports the AMEs based on the regression results from Tables 2, 3. The AMEs indicate that, on average, Black, Hispanic, and Asian adolescents were 7, 8, and 12 percentage points less likely (*p* < 0.001), respectively, to take prescription medication for mental health issues compared to White adolescents, controlling for other factors. Similar patterns were observed for receiving therapy and for receiving both medication and therapy. Black adolescents were also 8 percentage points less likely (*p* < 0.001) to have had any virtual care. In terms of community support, Hispanic and Asian adolescents were 9% and 15% points less likely (*p* < 0.001), respectively, to report having at least one adult, other than a parent or household member, in their school, neighborhood, or broader community who made a positive and meaningful difference in their life.

**Table 4:** Average marginal effects on mental healthcare utilization, virtual care, and social support among adolescents.

	**Took medication for mental health**				**Had therapy**				**Had medication and therapy**			
	**AME**	**95% CI**		***p***	**AME**	**95% CI**		***p***	**AME**	**95% CI**		***p***
**Race and ethnicity**												
White	reference				reference				reference			
Black	−0.07	−0.09	−0.06	< 0.001	−0.07	−0.09	−0.05	< 0.001	−0.04	−0.06	−0.03	< 0.001
Hispanic	−0.08	−0.09	−0.06	< 0.001	−0.04	−0.06	−0.03	< 0.001	−0.04	−0.06	−0.03	< 0.001
Asian	−0.12	−0.13	−0.10	< 0.001	−0.12	−0.14	−0.10	< 0.001	−0.07	−0.09	−0.06	< 0.001
	**Had any virtual care**				**Emotional/social support available**				**Presence of community support**			
	**AME**	**95% CI**		***p***	**AME**	**95% CI**		***p***	**AME**	**95% CI**		***p***
**Race and ethnicity**												
White	reference				reference				reference			
Black	−0.08	−0.11	−0.05	< 0.001	0.04	< 0.001	0.07	0.05	−0.02	−0.05	< 0.001	0.06
Hispanic	−0.01	−0.04	0.01	0.34	−0.03	−0.06	< 0.001	0.04	−0.09	−0.11	−0.07	< 0.001
Asian	−0.06	−0.09	−0.02	< 0.001	−0.06	−0.11	−0.02	0.01	−0.15	−0.20	−0.11	< 0.001

Data: Our study used the 2019–2022 National Health Interview Survey's (NHIS) Sample Child Interview and focused on adolescents aged 12–17. “Emotional/social support” measure was only available in 2021 and 2022, and “had virtual medical appointment” measure was only available since 2020. Other covariates were controlled.

## Discussion

The results of our study show that access to mental health services, virtual care, and community support remains disproportionately limited for racial and ethnic minority adolescents, even as overall mental health needs have worsened across all groups during the COVID-19 pandemic, a pattern that is consistent with the literature prior to the pandemic (9–11).

Using Andersen's Behavioral Model, we accounted for SES such as family income, parental education, and insurance coverage. As shown in our results, higher parental education and insurance coverage were generally associated with greater mental health service utilization. Nevertheless, even after controlling for these socioeconomic factors, we continued to observe significant racial and ethnic disparities, suggesting the influence of additional unmeasured factors. Cultural norms and stigma may play a key role. For example, mental health concerns are often underreported in minority communities due to cultural beliefs that discourage open discussion of emotional distress (19). Furthermore, racial and ethnic minority families may face structural barriers to accessing timely diagnosis and treatment for depression, including language barriers, healthcare navigation challenges, provider bias, and a lack of culturally competent care (20). Stigma related to depression has also been shown to vary by race and ethnicity, potentially contributing to underutilization of services (19–22).

Although we controlled for mental health needs, our analysis relied on a parent-reported proxy for depressive symptoms due to the absence of objective diagnostic data in the survey. It is important to note that the frequency of feeling depressed perceived by parents may not fully align with adolescents' lived experiences. To more accurately capture disparities and inform effective interventions, future research should incorporate culturally sensitive, adolescent-reported measures of mental health.

Community social support has been widely recognized as an important protective factor in promoting adolescent mental health (9, 23). For example, research has shown that social support can moderate the negative impact of depression on physical health among Hispanic youth (24). In our study, Hispanic and Asian adolescents were significantly less likely to report receiving community support. These findings are consistent with existing literature, which suggests that the mental healthcare system may not fully account for culturally influenced help-seeking behaviors among Hispanic and Asian American adolescents (25).

Literature also suggests that Hispanic and Asian American youth may be more inclined to rely on peers and express themselves more openly with friends, potentially creating pathways to informal support and reducing the stigma associated with seeking formal mental health services (26). These insights highlight the need for culturally responsive strategies in schools and communities. Peer support programs, culturally attuned counseling services, and mental health education initiatives may help improve recognition, communication, and access to care among these populations (24–26).

For example, school-based mental health services (SBMHS) offer a promising approach to improving access by embedding care within educational settings, reducing barriers related to stigma and availability (27). Additionally, expanding the peer support workforce—through programs that train individuals with lived experience to support children, adolescents, and their families—can foster trust, cultural relevance, and engagement in mental health care (14).

Racial and ethnic minority adolescents were less likely to have a virtual health appointment, despite the overall increase in telemedicine use during the study period (28, 29). Previous research has shown that Black and Hispanic families are less likely than White families to own a home computer or have reliable internet access (30). In our study, the telehealth measure included both phone and video visits. Studies have found that telehealth use among Black and Hispanic households often relies more heavily on phone calls, likely due to limited broadband access and other infrastructural barriers (31). These findings highlight the need for future research to focus specifically on access-related barriers to virtual care, including disparities in broadband infrastructure, device availability, and digital literacy.

Interestingly, although Asian families are more likely than White families to own a home computer and report higher rates of internet usage (32), Asian adolescents in our sample were significantly less likely to use virtual mental health care. This suggests that factors beyond technology access, such as limited mental health literacy, language barriers, or reluctance to seek help, may be contributing to lower virtual care use among Asian adolescents.

Although our primary focus is on mental health service utilization, we also note that our findings showed a worsening mental health status among adolescents, particularly among racial and ethnic minority groups, since 2019. Significant racial and ethnic disparities in access to mental health services persisted, with racial and ethnic minorities being more affected. These trends may be associated with the extended period of isolation due to the COVID-19 pandemic, which limited social interaction and increased exposure to social media and screen times, factors known to harm adolescent mental health, the increasing frequency of depression among race and ethnic minority adolescents cannot be overlooked (4, 5). Our findings indicated that the gap between White and minority adolescents who were depressed narrowed during the study period, highlighting the urgent need and practices (33, 34) to address mental health among racial and ethnic minority adolescents post the pandemic.

This study has important limitations. First, the cross-sectional design limits our ability to draw causal inferences; all observed associations should be interpreted as correlational. Although we controlled for a wide range of sociodemographic and contextual variables, unmeasured confounders, such as household dynamics, community-level resources, or experiences of discrimination, may still have influenced the results. Second, the measure of telemedicine use was broad and not specific to mental health services. It also did not distinguish between modalities such as video and telephone visits, differences that may carry important implications for accessibility, engagement, and effectiveness. Third, mental health measures were based on parent-reported proxies, which may underestimate the true prevalence of adolescent mental health issues due to stigma, social desirability bias, or limited parental awareness. Future research should further examine how racial and ethnic disparities in mental health care interact with socioeconomic status and other contextual factors. Moreover, the persistently low use of mental health services among minority adolescents may be driven by additional influences not captured in our model, including gaps in insurance coverage for mental health care, variation in actual mental health needs, barriers to both in-person and telehealth services, and culturally specific stigma surrounding mental illness. Understanding these complex and intersecting factors is essential to developing more equitable and culturally responsive mental health systems for adolescents.

## Conclusion

This study provides new evidence on racial and ethnic disparities in adolescent mental health service utilization, social support, and telemedicine use in the United States from 2019 to 2022. While mental health challenges have worsened for adolescents across all groups during this period, our findings show that Black, Hispanic, and Asian adolescents continue to face significantly lower odds of accessing mental health services compared to their White peers. These disparities persist even after controlling for SES and other access factors, underscoring the need to look beyond traditional barriers to care. The study also highlights important gaps in virtual care and community support. Despite tAhe growth in telemedicine, racial and ethnic minority adolescents were less likely to access virtual health services, pointing to ongoing issues of digital access, cultural stigma, and system-level barriers. Similarly, lower levels of reported community and emotional support among Hispanic and Asian adolescents suggest a need for culturally responsive support networks. To advance adolescent mental health for all, interventions must go beyond expanding access. Efforts should address cultural stigma, improve health literacy, and strengthen school- and community-based support systems. Future research should examine how cultural factors influence mental health service use and how policies can better support culturally tailored care for all adolescents.

## Data Availability

The original contributions presented in the study are included in the article/Supplementary material, further inquiries can be directed to the corresponding author.

## References

[1] National Institute of Mental Health. Transforming the Understanding and Treatment of Mental Illness, Major Depression. Available online at: https://www.nimh.nih.gov/health/statistics/major-depression (accessed May 6, 2025).

[2] Viner RM Ross D Hardy R Diana K Power C Johnon A. Life course epidemiology: recognizing the importance of adolescence. J Epidemiol Community Health. (2015) 69:719–20. 10.1136/jech-2014-205300 25646208 PMC4515995

[3] Rodgers CRR Flores MW Bassey O Augenblick JM Cook BL. Racial/ethnic disparity trends in children's mental health care access and expenditures from 2010–2017: disparities remain despite sweeping policy reform. J Am Acad Child Adolesc Psychiatry. (2022) 61:915–25. 10.1016/j.jaac.2021.09.420 34627995 PMC8986880

[4] Gotlib IH Miller JG Borchers LR Coury SM Costello LA Garcia JM. Effects of the COVID-19 pandemic on mental health and brain maturation in adolescents: implications for analyzing longitudinal data. Biol Psychiatry Glob Open Sci. (2022) 3:912–8. 10.1016/j.bpsgos.2022.11.002 36471743 PMC9713854

[5] Pettersen JH Hannigan LJ Gustavson K Lund IO Pearson RM Jensen P. COVID-19 pandemic quarantines and mental health among adolescents in Norway. JAMA Netw Open. (2024) 7:e2422189. 10.1001/jamanetworkopen.2024.22189 38995642 PMC11245726

[6] Acoba EF. Social support and mental health: the mediating role of perceived stress. Front Psychol. (2024) 15:1330720. 10.3389/fpsyg.2024.1330720 38449744 PMC10915202

[7] Barney A Mendez-Contreras S Hills NK Buckelew SM Raymond-Flesch M. Telemedicine in an adolescent and young adult medicine clinic: a mixed methods study. BMC Health Serv Res. (2023) 23:680. 10.1186/s12913-023-09634-x 37349720 PMC10288754

[8] Andersen RM. Revisiting the behavioral model and access to medical care: does it matter? J Health Soc Behav. (1995) 36:1–10. 10.2307/2137284 7738325

[9] Alegria M Vallas M Pumariega AJ. Racial and ethnic disparities in pediatric mental health. Child Adolesc Psychiatr Clin N Am. (2010) 19:759–74. 10.1016/j.chc.2010.07.001 21056345 PMC3011932

[10] Lu W Todhunter-Reid A Mitsdarffer ML Muñoz-Laboy M Yoon AS Xu L. Barriers and facilitators for mental health service use among racial/ethnic minority adolescents: a systematic review of literature. Front Public Health. (2021) 9:641605. 10.3389/fpubh.2021.641605 33763401 PMC7982679

[11] Martin R Banaag A Riggs DS Koehlmoos TP. Minority adolescent mental health diagnosis differences in a national sample. Mil Med. (2022) 187:e969–77. 10.1093/milmed/usab326 34387672

[12] Zhou W Franzini L. The role of social support in mitigating the effects of increased screen time on adolescent mental health. PLos Mental Health. (2025) 2:e0000213. 10.1371/journal.pmen.0000213 PMC12798297 41661911

[13] Panchal U Salazar de Pablo G Franco M Moreno C Parellada M Arango C. The impact of COVID-19 lockdown on child and adolescent mental health: systematic review. Eur Child Adolesc Psychiatry. (2023) 32:1151–1177. 10.1007/s00787-021-01856-w 34406494 PMC8371430

[14] Robertson HA Biel MG Hayes KR Snowden S Curtis L Charlot-Swilley D. Leveraging the expertise of the community: a case for expansion of a peer workforce in child, adolescent, and family mental health. Int J Environ Res Public Health. (2023) 20:5921. 10.3390/ijerph20115921 37297524 PMC10252488

[15] U.S. Department of Health and Human Services. Healthy People 2030. Office of Disease Prevention and Health Promotion. Available online at: https://health.gov/healthypeople (accessed May 6, 2025).

[16] National Center for Health Statistics. National Health Interview Survey, 2019–2022: Sample Child Files (2023). Available online at: https://www.cdc.gov/nchs/nhis/documentation/2022-nhis.html (accessed May 6, 2025).

[17] National Center for Health Statistics. 2022 National Health Interview Survey (NHIS) Survey Description. U.S. Department of Health and Human Services, Centers for Disease Control and Prevention (2023). Available online at: https://ftp.cdc.gov/pub/Health_Statistics/NCHS/Dataset_Documentation/NHIS/2022/srvydesc-508.pdf (accessed May 6, 2025).

[18] Santos RMS Mendes CG Sen Bressani GY de Alcantara Ventura S de Almeida Nogureira YJ de Miranda DM. The associations between screen time and mental health in adolescents: a systematic review. BMC Psychol. (2023) 11:127. 10.1186/s40359-023-01166-7 37081557 PMC10117262

[19] DuPont-Reyes MJ Villatoro AP Phelan JC Painter K Link BG. Adolescent views of mental illness stigma: an intersectional lens. Am J Orthopsychiatry. (2020) 90:201–11. 10.1037/ort0000425 31380669 PMC7000296

[20] Misra S Jackson VW Chong J Choe K Tay C Wong J. Systematic review of cultural aspects of stigma and mental illness among racial and ethnic minority groups in the United States: implications for interventions. Am J Community Psychol. (2021) 68:486–512. 10.1002/ajcp.12516 33811676

[21] Eboigbe LI Simon CB Wang YS. Tyrell FA. The compounded effect of the dual pandemic on ethnic-racial minority adolescents' mental health and psychosocial well-being. Curr Opin Psychol. (2023) 52:101626. 10.1016/j.copsyc.2023.101626 37384949 PMC10293782

[22] Williams DR Priest N Anderson NB. Understanding associations among race, socioeconomic status, and health: patterns and prospects. Health Psychol. (2016) 35:407–11. 10.1037/hea0000242 27018733 PMC4817358

[23] Buitron V Jiménez-Colón G Duarté-Vélez Y. Mental health services use and social support among Latinx families with adolescents who engage in suicidal behavior. Evid Based Pract Child Adolesc Ment Health. (2023) 8:194–205. 10.1080/23794925.2023.2183433 37383484 PMC10299760

[24] Cariello AN Perrin PB Williams CD Espinoza GA Paredes AM Moreno OA. Moderating influence of social support on the relations between discrimination and health via depression in Latinx immigrants. J Lat Psychol. (2022) 10:98–111. 10.1037/lat0000200 35434535 PMC9012262

[25] Chang J Chen C-H Alegría M. Contextualizing social support: pathways to help seeking in Latinos, Asian Americans, and Whites. J Soc Clinical Psychol. (2014) 33:1–24. 10.1521/jscp.2014.33.1.1

[26] Lu S Hart LM Jorm AF Gregg K Gross M Mackinnon AJ. Adolescent peer support for mental health problems: evaluation of the validity and reliability of the mental health support scale for adolescents. BMC Psychol. (2023) 11:193. 10.1186/s40359-023-01228-w 37391834 PMC10314445

[27] Richter A Sjunnestrand M Romare Strandh M Hasson H. Implementing school-based mental health services: a scoping review of the literature summarizing the factors that affect implementation. Int J Environ Res Public Health. (2022) 19:3489. 10.3390/ijerph19063489 35329175 PMC8948726

[28] Evans YN Golub S Sequeira GM Eisenstein E North S. Using telemedicine to reach adolescents during the COVID-19 pandemic. J Adolesc Health. (2020) 67:469–71. 10.1016/j.jadohealth.2020.07.015 32768330 PMC7403159

[29] Gilkey M Kong W Huang Q Grabert B Thompson P Brewer N. Using telehealth to deliver primary care to adolescents during and after the COVID-19 pandemic: national survey study of US primary care professionals. J Med Internet Res. (2021) 23:e31240. 10.2196/31240 34406974 PMC8437399

[30] Fairlie RW. Are we really a nation online? Ethnic and racial differences in access to technology and possible explanations. Report for the Leadership Conference on Civil Rights Education Fund (2005).

[31] Benjenk I Franzini L Roby D Chen J. Disparities in audio-only telemedicine use among Medicare beneficiaries during the coronavirus disease 2019 pandemic. Med Care. (2021) 59:1014–22. 10.1097/MLR.0000000000001631 34534186 PMC8516710

[32] Liu JL Wang C Do KA Bali D. Asian American adolescents' mental health literacy and beliefs about helpful strategies to address mental health challenges at school. Psychol Sch. (2022) 59:2062–84. 10.1002/pits.22655

[33] Ialongo N Poduska J Werthamer L Kellam S. The distal impact of two first-grade preventive interventions on conduct problems and disorder in early adolescence. J Emot Behav Disord. (2001) 9:146. 10.1177/106342660100900301

[34] Kataoka SH Stein BD Jaycox LH Wong M Escudero P Tu W. A school-based mental health program for traumatized Latino immigrant children. J Am Acad Child Adolesc Psychiatry. (2003) 42:311–8. 10.1097/00004583-200303000-00011 12595784

