# Supplementary material for: Adolescent mental health utilization, virtual care, and community support: evidence from 2019 to 2022
Source: Front Public Health. 2025 Jul 28;13:1559511. doi: 10.3389/fpubh.2025.1559511 (PMC12337129; doi:10.3389/fpubh.2025.1559511)
Supplement: Supplementary file 1 [file Supplementary_file_1.docx]

**Appendix Table 1: Regression results not controlling for parent reported frequency of feeling depressed**

|  | **Took medication for mental health** | | | | **Had therapy** | | | | **Had medication and therapy** | | | |
| --- | --- | --- | --- | --- | --- | --- | --- | --- | --- | --- | --- | --- |
|  | OR | 95% CI | | p | OR | 95% CI | | p | OR | 95%CI | | p |
| *Race and ethnicity* | | | | | | | | | | | | |
| White | reference | | | | reference | | | | reference | | | |
| Black | 0.30 | 0.19 | 0.47 | <0.001 | 0.55 | 0.36 | 0.82 | <0.001 | 0.37 | 0.21 | 0.63 | <0.001 |
| Hispanic | 0.39 | 0.28 | 0.54 | <0.001 | 0.66 | 0.50 | 0.87 | <0.001 | 0.54 | 0.37 | 0.79 | <0.001 |
| Asian | 0.16 | 0.07 | 0.34 | <0.001 | 0.22 | 0.12 | 0.38 | <0.001 | 0.21 | 0.09 | 0.51 | <0.001 |
|  | **Had any virtual care** | | | | **Emotional/social support available** | | | | **Presence of community support** | | | |
|  | OR | 95% CI | | p | OR | 95% CI | | p | OR | 95% CI | | p |
| *Race and ethnicity* | | | | | | | | | | | | |
| White | reference | | | | reference | | | | reference | | | |
| Black | 0.59 | 0.36 | 0.96 | 0.04 | 1.34 | 0.90 | 2.00 | 0.15 | 1.06 | 0.49 | 2.28 | 0.88 |
| Hispanic | 0.82 | 0.62 | 1.09 | 0.17 | 1.08 | 0.81 | 1.45 | 0.60 | 0.27 | 0.17 | 0.42 | <0.001 |
| Asian | 0.63 | 0.39 | 1.04 | 0.07 | 0.75 | 0.49 | 1.13 | 0.17 | 0.30 | 0.16 | 0.57 | <0.001 |

Note: Data: Our study used the 2019-2022 National Health Interview Survey’s (NHIS) Sample Child Interview and focused on adolescents aged 12 to 17. “Emotional/social support” measure was only available in 2021 and 2022, and “had virtual medical appointment” measure was only available since 2020. Logistic regressions were applied, and other covariates were controlled.

**Appendix Table 2: Regression results controlling for screen-time**

|  | **Took medication for mental health** | | | | **Had therapy** | | | | **Had medication and therapy** | | | |
| --- | --- | --- | --- | --- | --- | --- | --- | --- | --- | --- | --- | --- |
|  | OR | 95% CI | | p | OR | 95% CI | | p | OR | 95%CI | | p |
| *Race and ethnicity* | | | | | | | | | | | | |
| White | reference | | | | reference | | | | reference | | | |
| Black | 0.36 | 0.22 | 0.58 | <0.001 | 0.69 | 0.45 | 1.06 | 0.09 | 0.47 | 0.26 | 0.83 | 0.01 |
| Hispanic | 0.45 | 0.32 | 0.62 | <0.001 | 0.80 | 0.61 | 1.06 | 0.13 | 0.64 | 0.44 | 0.95 | 0.03 |
| Asian | 0.16 | 0.07 | 0.34 | <0.001 | 0.21 | 0.12 | 0.37 | <0.001 | 0.22 | 0.09 | 0.52 | <0.001 |
|  | **Had any virtual care** | | | | **Emotional/social support available** | | | | **Presence of community support** | | | |
|  | OR | 95% CI | | p | OR | 95% CI | | p | OR | 95% CI | | p |
| *Race and ethnicity* | | | | | | | | | | | | |
| White | reference | | | | reference | | | | reference | | | |
| Black | 0.67 | 0.40 | 1.11 | 0.12 | 1.18 | 0.79 | 1.76 | 0.41 | 1.07 | 0.49 | 2.32 | 0.87 |
| Hispanic | 0.91 | 0.69 | 1.22 | 0.54 | 0.95 | 0.71 | 1.28 | 0.74 | 0.27 | 0.17 | 0.42 | <0.001 |
| Asian | 0.65 | 0.39 | 1.09 | 0.11 | 0.71 | 0.46 | 1.09 | 0.12 | 0.30 | 0.16 | 0.57 | <0.001 |

Note: Data: Our study used the 2019-2022 National Health Interview Survey’s (NHIS) Sample Child Interview and focused on adolescents aged 12 to 17. “Emotional/social support” measure was only available in 2021 and 2022, and “had virtual medical appointment” measure was only available since 2020. Logistic regressions were applied, and other covariates were controlled.
